# Synthesizing Depth Hand Images with GANs and Style Transfer for Hand Pose Estimation

**DOI:** 10.3390/s19132919

**Published:** 2019-07-01

**Authors:** Wangyong He, Zhongzhao Xie, Yongbo Li, Xinmei Wang, Wendi Cai

**Affiliations:** School of Automation, China University of Geosciences, Wuhan 430074, China

**Keywords:** hand pose estimation, generative adversarial networks, style transfer, human-computer interaction, depth images

## Abstract

Hand pose estimation is a critical technology of computer vision and human-computer interaction. Deep-learning methods require a considerable amount of tagged data. Accordingly, numerous labeled training data are required. This paper aims to generate depth hand images. Given a ground-truth 3D hand pose, the developed method can generate depth hand images. To be specific, a ground truth can be 3D hand poses with the hand structure contained, while the synthesized image has an identical size to that of the training image and a similar visual appearance to the training set. The developed method, inspired by the progress in the generative adversarial network (GAN) and image-style transfer, helps model the latent statistical relationship between the ground-truth hand pose and the corresponding depth hand image. The images synthesized using the developed method are demonstrated to be feasible for enhancing performance. On public hand pose datasets (NYU, MSRA, ICVL), comprehensive experiments prove that the developed method outperforms the existing works.

## 1. Introduction

As human-computer interaction [1,2] has been optimized, a computer-vision method has been adopted to detect the 3D pose of the human hand and its knuckles from an image or image sequence in a non-contact manner. Thus, a machine can visually understand the 3D state and behavior of the human hand as a human would, making hand pose estimation [3,4] vital to human–computer interaction and augmented reality. By learning and analyzing the 3D motion state of human hands, it is possible to create a more natural and efficient human-computer interaction environment.

In recent years, based on a fully supervised convolutional neural network [5], some progress has been made in hand pose estimation using data-driven methods [6,7,8,9]. Many issues remain unresolved, since the human hand has the following characteristics [10,11,12]: multiple degrees of freedom, self-occlusion, and self-similarity in image. There are often only a limited number of manually annotated depth images, in which field experts portray hand joints through strenuous and time-consuming manual processes.

For data annotation, it is far too complicated and time-consuming to annotate 3D hand joints in depth images accurately. Although image synthesis can be generated using a physical renderer, there is usually a few differences between real and synthetic data, without consideration of depth sensor noise in a realistic way. Therefore, image synthesis has become a key data augmentation technique in terms of the latest developments in human or hand pose estimation, in which synthetic data plays an important role in solving classification and regression tasks. Meanwhile, there has been much research [13,14,15] into hand pose estimation, yet there are few works aimed at improving the accuracy of pose estimation by using a synthetic image for data augmentation.

In this paper, a data-driven method [16,17,18] is proposed for generating deep hand images closer to real ones during training. The developed method makes the following significant contribution: (1) the developed generation model learns from relatively limited training samples as driven by data, which helps conclude the mapping relationship between the hand pose and the corresponding depth image; (2) the generator networks of GAN significantly augment the training set and enhance hand pose estimation accuracy; (3) the discrepancies are reduced between real and synthesized data using style transfer to simulate hand noise in real-world environments. The proposed approach is evaluated based on three public hand pose benchmarks [4,19,20] (NYU, MSRA, ICVL). The developed method, compared to the existing excellent methods, achieves better performance on specific evaluation metrics.

The rest of the paper is organized as follows. In Section 2, previous works relevant to the developed method are reviewed. In Section 3, details about the proposed network structure to generate depth hand images are presented. In Section 4 and Section 5, experimental details and evaluations of public datasets are provided.

## 2. Related Works

Generative adversarial networks: The data distribution of an unlabeled training image is relatively difficult to learn through a neural network, and the prediction result cannot be achieved by such a data distribution, which is hard to calculate. With the rapid development of deep-learning techniques [21,22,23], the models to generate deep hand images have been proposed, such as the generative adversarial network (GAN). The GAN synthetic image method aims to achieve a Nash balance through the generative network and the discriminator network to play a zero-sum game. The discriminator distinguishes the real image from the composite image, while the generator aims to deceive the discriminator by approximating the real data distribution to generate the image. In perfect equilibrium [22], the generator is able to learn the real data distribution of the training data. In the meantime, the discriminator can always correctly distinguish between real and fake ones.

Subsequently, several works about GANs variants [24,25,26,27,28] have been extended to cover multiple domains (e.g., image synthesis) from image domain. Most recent GAN research focused on improving the quality and utility of image synthesis. Among them, the DCGAN [24] model exploits a constraint relationship to maintain the dynamic stability of the training between the generator and the discriminator. The CGAN [25] model helps train synthetic models to generate images based on ancillary information. The LAPGAN [26] model generates images and improves their quality from coarse to fine ones by using a cascade convolution network within the Laplacian pyramid framework. The InfoGAN [27] model helps learn in a completely unsupervised manner. The WGAN [28] model is easy to train, and it can use different objective functions, which are less sensitive to the nonlinear choices applied between convolutional layers. The generative depth map, i.e., synthesizing depth maps from given poses, should be realistic, because synthesis images are needed to train the entire network.

Image-style transfer, and data augmentation: Many scholars have discussed the problem of image-style transfer [29,30] in the past two decades. Recently, Gates et al. [31] achieved remarkable results by successfully using deep-learning technology. Many of the relevant works (e.g., [32,33]) have been further improved, which prioritizes efficiency and light weight. Current methods enrich existing training examples for data augmentation with style transfer. Moreover, the discrepancies between the real and synthesized data can decrease, in combination with style transfer, to model depth sensor noise realistically. Furthermore, the limited training samples can be enriched by scaling, cropping, translating, and rotating limited training images for data augmentation [34,35].

Hand pose estimation: The discriminating method directly learns from the labeled training data and then predicts the result. The predictive model can predict the probability map (heatmap) of each hand joint [2,36] or predict the 3D hand joint coordinates [37,38]. Random forests [19,20,39,40] and convolutional neural networks [41] are the most commonly used predictive models. The discriminant-based method aims to learn the mapping between the depth image and the 3D pose of the human hand. Such a data-driven method primarily trains the deep neural network through considerable human hand images with human hand 3D pose coordinates to achieve approximate model fitting. Many new methods are used to estimate hand poses by introducing feature extraction and 3D hand pose coordinate regression into the end-to-end learning framework. To achieve higher accuracy, much data is required to train networks, thus making manual tagging data highly expensive. As a result, a few approaches have been proposed to use the distribution of unlabeled depth maps or hand poses to learn better representations. Bouchacourt et al. [42] (DISCO) proposed a probabilistic framework. They employed a neural network to learn the posterior distribution of the human hand image and sample it from the distribution. However, these samples still cannot be combined practically. Wan et al. [41] proposed the VAE and GAN networks (Crossing Nets) to estimate hand pose. VAE is adopted to generate hand poses, GAN is used to synthesize deep hand image, and the whole network structure can learn the shared mapping between the two parts. The whole network model should be trained in complex ways. The work by Baek et al. [43] aimed to synthesize data in the skeleton space. Specifically, they combined the hand pose generator and estimator to exploit both existing paired skeletons and depth map entries and newly synthesized depth maps in a single unified framework. Oberweger et al. [44] developed the feedback loop network to estimate hand pose. The discriminative network is employed to generate the initial hand pose, which can be used to generate depth image by generative CNN. Subsequently, the generated images and real images are transferred to the shared convolutional neural network for hand pose estimation. Lastly, the initial hand pose can be continuously updated based on the update network. However, the depth image synthesis network is highly sensitive to the mislabeling of hand poses. The closest work [41] also uses techniques similar to GAN to generate depth hand image in the data augmentation process. Unlike [41], which tends to generate a depth hand image for a given hand pose, the developed method can create infinite phantoms from different style images with the same hand pose. This paper builds the correlation between depth image and hand pose through considerable synthetic and real images.

## 3. Synthesizing Depth Hand Images with GANs and Style Transfer

In the developed method, the major goal is to build a mapping relationship between hand pose and depth hand image based on a neural network. To be more specific, $x\in R^{W\times H}$ denotes a depth hand image, $y\in R^{3\times J}$ is hand poses. Then, the generation network is adopted to convert the hand pose into a deep hand image set $G_{\theta }:\left(y\in R^{3\times J}\right)$ produce $\bar{x}\in R^{W\times H}$. Our goals are three-fold: (1) to discover the probability distribution $p(\bar{x}|y)$ of deep hand images under the condition of hand pose *y*, where $\bar{x}$ denotes synthesized depth hand image conditioning on hand pose *y*; (2) the specific noise features in the style images $x_{s}$ with different noises are transferred to the smooth synthetic image to generate an image $\bar{x}$ closer to the real depth hand image; and (3) to demonstrate that the synthetic images effectively increase samples and improve the pose estimation accuracy.

To generate the depth hand images as similar to raw depth hand images as possible, we propose to combine GAN and style transfer to generate the synthetic images. The structure can be split into three parts (the generator, discriminator, and style-transfer network). The generator generates synthesized hand images with hand poses. Moreover, we follow the GAN idea of double zero-game settings and consider the optimization problem between the discriminator *G* and the generator *D*. The style-transfer network aims to transform the smooth synthetic images to become depth hand images more similar to real ones.

### 3.1. Generator G and Discriminator D

The generator is denoted as $G_{\theta }$ and the discriminator is expressed as $D_{r}$, then a zero-sum game between the generator *G* and the discriminator *D* is performed according to the GAN idea [22] to reach the Nash equilibrium point:

(1) $$\begin{array}{cc} \min_{\theta }\max_{r}L\left(G_{\theta },D_{r}\right)= & E_{x,y∼p(x,y)}\left[\log D_{r}\left(x,y\right)\right]\\ & +E_{y∼p(y)}\left[\log\left(1-D_{r}\left(G_{\theta }\left(y\right)\right)\right)\right]\\ & +\lambda L_{recons}\left(G_{\theta }\left(y\right)\right) \end{array}$$

λ is empirically set to 1, and the last item $\lambda L_{recons}\left(G_{\theta }\left(y\right)\right)$ is introduced to ensure that the synthesized images do not deviate away from the real hand images. The clipping mean square error loss function is adopted to ensure the robustness to the depth sensor noise. To facilitate the training of the model, the pixel values are normalized to [−1,1] in the depth map, and the threshold is set to τ=1, controlling how much noise is retained. *N* denotes the batch size. The following formulation is considered:

(2) $$L_{recons}\left(G_{\theta }\left(y\right)\right)=\frac{1}{N}\sum_{i}^{N}\max(||x^{\left(i\right)}-G_{\theta }\left(y^{\left(i\right)}\right){\left|\right|}^{2},\tau )$$

Given the depth of the depth hand image, the 3D hand pose can be estimated. To improve the discriminator’s ability to distinguish the synthesis image, the update parameter is expressed as $\theta_{pose}$. A loss function between annotated hand pose and prediction of hand pose is yielded as:

(3) $$L_{pose}=\frac{1}{N}\sum_{i}^{N}\left|\right|D_{\theta }\left(x^{\left(i\right)}\right)-y^{\left(i\right)}{\left|\right|}^{2}$$

In brief, the generator *G* can be trained to generate realistic depth hand images deceiving discriminator by minimizing Equation (1). In fact, the scheme of [22] suggests that we can train the generator by minimizing $-log(D_{r}\left(G_{\theta }\left(y\right)\right))$ instead of $log(1-D_{r}\left(G_{\theta }\left(y\right)\right))$. Thus, the training generator G is equivalent to minimizing the following formulation:

(4) $$L_{G}\left(G_{\theta }\right)=-\sum_{i}\log D_{r}\left(G_{\theta }\left(y_{i}\right)\right)+\lambda L_{recons}\left(G_{\theta }\left(y\right)\right)$$

Moreover, the discriminator *D* correctly distinguishes the synthetic depth hand image from the real image by maximizing the Equation (1). Thus, the discriminator *D* is trained to be equivalent to maximizing the following formulation:

(5) $$L_{D}\left(D_{r}\right)=\sum_{i}\log D_{r}\left(x_{i},y_{i}\right)+\log\left(1-D_{r}\left(G_{\theta }\left(y_{i}\right)\right)\right)$$

The loss function of the GAN network model is written as:

(6) $$L_{gan}=\log D_{r}\left(x_{i},y_{i}\right)+\log\left(1-D_{r}\left(G_{\theta }\left(y_{i}\right)\right)\right)$$

Accordingly, the joints loss function can be expressed as the generator loss function $L_{Gen}$, where the optimization parameter is $\theta_{Gen}$ and the discriminator loss function is $L_{Dis}$, where the optimization parameter is $\theta_{Dis}$:

(7) $$L_{Gen}=L_{recons}-L_{gan}$$

(8) $$L_{Dis}=L_{pose}+L_{gan}$$

The learning process of the GAN network is achieved through the alternating optimization of $L_{Gen}$ and $L_{Dis}$. However, this optimization procedure does not achieve the formal guarantee of Nash Equilibrium. Next, the specific architecture of the function *G* and *D* combined with the style-transfer network is detailed.

### 3.2. The Style-Transfer Variant

Style transfer [29,30,31] is applied to generate the image, of which style is equivalent to a style image, and the content is equal to the content image. To define a style and content representation clearly, a loss function can be defined, which shows us how far away our synthesized images are from the perfect style transfer.

Without style transfer, the synthetic images from the generator are rather smooth, so style transfer can be applied to make the synthetic images more similar to real ones. We hold the idea of style transfer, and employ VGG-19 [45] convolutional neural network to extract content features and style features from multiple convolutional layers. The index of the layer *i* and the index of the block *j* can be defined. Next, the architecture of our style-transfer specific network is detailed.

#### 3.2.1. Content Loss

Given the chosen content layer *l*, the content loss is defined as the Euclidean distance between the feature map $F^{l}$ of our content image *x* and the feature map $P^{l}$ of our generated image $\hat{x}$. When the content representation of image *C* is identical to that of image *Y*, the loss is approximately 0:

(9) $$L_{cont}\left(G_{\theta }\right)=\sum_{i,j}\frac{1}{2}{\left(F_{i,j}^{l}-P_{i,j}^{l}\right)}^{2}$$

#### 3.2.2. Style Loss

We will do something similar for the style layers, where the features in the style layers activated simultaneously for the style image are measured, and then this activation pattern is copied to the mixed image. These feature correlations are given by Gram matrix $G_{i,j}^{l}$, where $G_{i,j}^{l}$ denotes the inner product between the vectorized feature map *i* and *j* in layer *l*:

(10) $$G_{i,j}^{l}=\sum_{k}F_{i,k}^{l}F_{j,k}^{l}$$

The loss function for style is significantly similar to our content loss, except that the Mean Squared Error for the Gram-matrices is calculated, instead of the raw tensor outputs from the layers.

(11) $$L_{sty}\left(G_{\theta }\right)=\frac{1}{2}\sum_{l=0}{\left(G_{i,j}^{l}-A_{i,j}^{l}\right)}^{2}$$

#### 3.2.3. Total Variation Loss

Furthermore, by combining the following total variation losses ($\bar{x}$ for generated phantoms, $\bar{x}\in R^{W\times H}$), we encourage spatial smoothing in synthesized depth hand image.

(12) $$L_{tv}\left(G_{\theta }\right)=\sum_{w,h}\left(|\right|{\bar{x}}_{w,h+1}-{\bar{x}}_{w,h}{\left|\right|}_{2}^{2}+\left|\right|{\bar{x}}_{w+1,h}-{\bar{x}}_{w,h}{\left|\right|}_{2}^{2})$$

the image size of w,h∈W,H, and $x^{w,h}$ denotes the pixel value of the given position in the generated image $\bar{x}$.

(13) $$L_{ST}\left(G_{\theta }\right)=w_{cont}L_{cont}+w_{sty}L_{sty}+w_{tv}L_{tv}$$

The total loss value of the style-transfer network covers content loss, style loss, as well as variation loss, where $w_{cont}$, $w_{sty}$, $w_{tv}$ denote the weight of $L_{cont}$, $L_{sty}$, $L_{tv}$, respectively

(14) $$L_{G}\left(G_{\theta }\right)=-\sum_{i}\log D_{r}\left(G_{\theta }\left(y_{i}\right)\right)+L_{ST}\left(G_{\theta }\right)$$

Since the style-transfer network acts as part of the generator to encourage the generator to generate realistic depth hand images, the generator’s optimization function becomes the following formulation:

Algorithm 1 suggests that the whole algorithm model first generates a smooth depth hand image by the generator of the generative adversarial network; subsequently, the style-transfer network is employed to introduce the hand noise in the real environment, to generate a more realistic depth image; lastly, synthesis images are inputted to GAN discriminator to determine how far the generated images are from the real ones. In the meantime, hand poses can be estimated through the residual network similar to ResNet-50 [46]. The balance is reached by the two-player zero-game between the generator and the discriminator.

**Algorithm 1.** Generate depth hand image via GAN and Style Transfer
1:$\theta_{Gen},\theta_{Dis}$ initialized through pretraining2:$\theta_{pose}$ randomly initialized3:
$\theta_{G}:=\theta_{Gen}$
4:
$\theta_{D}:=\theta_{pose}\cup \theta_{Dis}$
5:for number of training epoch do6:     x,y paired depth image and hand pose7:    $L_{recons}\left(G_{\theta }\left(y\right)\right)=\frac{1}{N}\sum_{i}^{N}\max(||x^{\left(i\right)}-G_{\theta }\left(y^{\left(i\right)}\right){\left|\right|}^{2},\tau )$
8:    $L_{pose}=\frac{1}{N}\sum_{i}^{N}\left|\right|D_{\theta }\left(x^{\left(i\right)}\right)-y^{\left(i\right)}{\left|\right|}^{2}$9:    $L_{cont}\left(G_{\theta }\right)=\sum_{i,j}\frac{1}{2}{\left(F_{i,j}^{l}-P_{i,j}^{l}\right)}^{2}$
10:    $L_{sty}\left(G_{\theta }\right)=\frac{1}{2}\sum_{l=0}{\left(G_{i,j}^{l}-A_{i,j}^{l}\right)}^{2}$
11:    $L_{tv}\left(G_{\theta }\right)=\sum_{w,h}\left(|\right|{\bar{x}}_{w,h+1}-{\bar{x}}_{w,h}{\left|\right|}_{2}^{2}+\left|\right|{\bar{x}}_{w+1,h}-{\bar{x}}_{w,h}{\left|\right|}_{2}^{2})$
12:    $L_{ST}\left(G_{\theta }\right)=w_{cont}L_{cont}+w_{sty}L_{sty}+w_{tv}L_{tv}$13:    $L_{gan}=\frac{1}{N}\sum_{1}^{N}||\log(D\left(x^{\left(i\right)},y^{\left(i\right)}\right)+\log(1-D\left(G\left(y^{\left(i\right)}\right)\right){\left|\right|}^{2}+L_{ST}\left(G_{\theta }\right)$14:    $L_{Gen}=L_{recons}-L_{gan}$15:    $L_{Dis}=L_{pose}+L_{gan}$16:    $\theta_{D}\leftarrow \theta_{D}-\nabla_{\theta_{D}}\left(L_{pose}-L_{gan}\right)$
17:    $\theta_{G}\leftarrow \theta_{G}-\nabla_{\theta_{G}}\left(L_{recons}+L_{gan}\right)$
18:end for


## 4. Experimental Details

### 4.1. Datasets and Preparation

Empirically, the developed method is examined on three different standard datasets (NYU, MSRA, and ICVL depth hand pose datasets). These datasets exhibit different image sizes and numbers of training samples: NYU contains 72,757 training samples and 8252 test images, with an image size of 480×640. The MSRA dataset contains 76500 training images, as well as the split of training/testing images being of size 240×320. The ICVL dataset is considered here for hand pose estimation, which contains 330,000 training images and 1596 test images of size 240×320.

To sum up, the depth images of NYU, MSRA, and ICVL are similar. When preprocessing depth hand image, the original image size of these datasets is adjusted to 128×128. As shown below, for the NYU dataset, all depth images are the size 480×640, which covers a relatively broad background area. Thus, the image should be cropped to a 128×128 sub-image centered on the original image, ensuring that all hand pixels remain in the cropped image. The bicubic interpolation is applied to further 128×128 adjustment. For MSRA and ICVL hand datasets, the images have relatively small background margins outside their hand masks. They are also cropped to 128×128 to preserve sufficient hand information for the original image, and the pixel values of all input images are normalized to scale to [−1,1]. Several good practices that have proved quite feasible to estimate hand poses are followed. For data augmentation, random scaling of [0.9,1.1], random translation of [−5,5] pixels, and random rotation of $\left[-180^{\circ },180^{\circ }\right]$ degrees to depth image are applied.

### 4.2. Model Architecture and Internal Parameters

**Figure 1:**
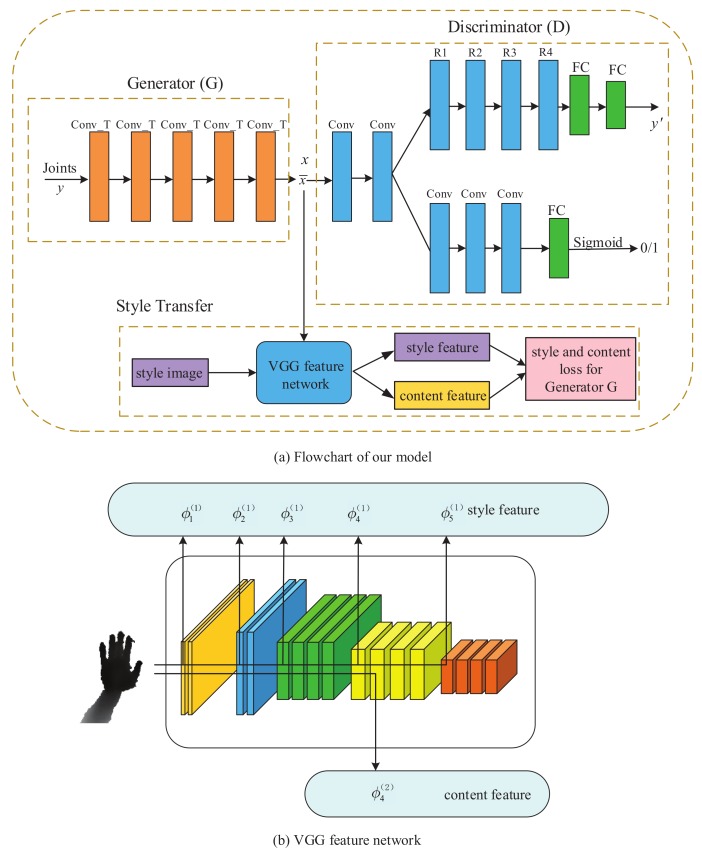
(**a**) Flowchart of the proposed method, covering the generator, the discriminator, and style-transfer networks in detail. The VGG-19 feature networks are described in (**b**), where the top row represents the style layers (e.g., $\phi_{1}^{1},\phi_{2}^{2},\ldots$ ) and the bottom row represents the content features (e.g., $\phi_{4}^{2}$). See text for details.

Figure 1a suggests that the architectural structure of the developed method is detailed: In the generator and discriminator modules, each rectangle represents the CNN layer including its feature map. To be specific, the synthesized image $\bar{x}$ is generated through the continuous deconvolutional operation. The deconvolution filter size is 5×5, yielding a depth image of size 128×128. The entire generative model of depth hand image consists of three parts: the first part is the generator of the GAN, transforming the 3D hand pose into a deep hand image; the second part acts as the discriminator of the GAN, determining the authenticity of synthesis image; the third part is performs 3D hand pose regression on depth hand images based on the residual convolutional neural network. In Figure 1a, *y* denotes the 3D hand pose (3D coordinates of hand joints), Conv_T is deconvolution layer, deconvolution kernel size is 6×6, deconvolution kernel channel is 32, dilation factor is 2, Conv stands for the convolution layer, which has a convolution kernel size of 6×6, and convolutional kernel is 32 channels, and a step size of 2. FC is a fully connected layer. To prevent over-fitting of the model, the model parameters are reduced by sharing the first layer and the second layer convolution network of the discriminator. Besides, the model convergence can be accelerated.

Through the experiment of the developed method, the internal parameters of the model are adjusted based on experience: the TensorFlow deep-learning framework is used for training. First, the training period is set to 100, and then the discriminator’s neural network weights *D* and the generator’s neural network weights *G* (i.e., parameter θ, *r*) are initialized. We use truncated normal distribution from minus 0.01 to 0.01, and the standard deviation is 0.01. Lastly, we set the batch size to 32 and use the Adam optimizer to update the *G* weight to θ, while use the stochastic gradient descent optimizer to update the *D* weight to *r*. During the backpropagation of model training, the learning rate of the generator is set to 0.0005, and the learning rate of the discriminator is set to 0.0003. To balance the learning speed of both *G* and *D*, in each iteration of the update, we update *D* twice and update *D* once. In our style-transfer network, the VGG-19 network extracts the style of a style image and the content of a content image for mixing to generate the depth hand image. Some layers in the network structure are adopted to extract style and content features, as shown in Figure 1b, the style index set is $\Gamma_{s}=1,2,3,4$ and the content index $\Gamma_{c}=4$. In the meantime, the weights of the three corresponding loss functions are expressed as $w_{Cont}$, $w_{sty}$, $w_{tv}$, with coefficients of 1, 5, and 50, respectively.

For our improved residual networks, numerous studies have been conducted on deep neural networks [9,34] and residual networks [46]. The residual network is the best existing performance model. Our proposed network structure for hand pose regression is similar to the 50-layer residual network (ResNet-Hand) model [46]. Since the residual network has achieved breakthrough results in image classification of ImageNet datasets, the network can be optimized as a model for human pose regression. The specific step is to remove the global average pooling layer, then add two fully connected layers for regressing the hand poses. The input depth hand image is the size of 128×128 and normalizes the pixels from minus 1 to 1. As shown in Figure 1, the improved ResNet model consists of two shared convolutional layers, 32 filters and 2×2 max-pooling, followed by four consecutive residual modules, including 64, 128, 256, 256 filter and stride of 2×2. Lastly, the last two fully connected layers are employed to estimate the hand pose coordinates, with the dropout set to 0.5 to avoid over-fitting of the model.

All experiments were performed on a server with an Intel iCore 7 CPU, 64 GB RAM, and a GTX1080TI GPU with 11 GB of RAM. Our GAN and Style-Transfer networks are implemented in TensorFlow. The training time in different data sets (NYU, MSRA, ICVL) was approximately 12 h, 9 h, and 7 h, respectively. Using a robust data augmentation method, the averages running time of the synthesized depth hand image is nearly 0.4633 s.

## 5. Empirical Experiments

The two different metrics are adopted to evaluate the developed method of hand pose estimation on the three public datasets: the joints mean error and the fraction of frames over maximum allowed distance to ground truth. $\left(X_{ij},Y_{ij},Z_{ij}\right)$ denotes the predicted joint locations of test frames, where *i* is the index of frame and *j* is the index of joint. $\left(X_{ij}^{gt},Y_{ij}^{gt},Z_{ij}^{gt}\right)$ is the corresponding ground-truth label. *N* is the number of test frames and *J* is the number of joints in a frame.

*The Average 3D Joint Error:* In the test set, we quantitatively calculate the average Euclidean distance between the 3D coordinates of each joint and the ground truth, and the average error of all joints on the all test frames is calculated by the following formula:

(15) $$err=\frac{1}{N}\sum_{i}\frac{1}{J}\sum_{j}\sqrt{{\left(X_{ij}^{gt}-X_{ij}\right)}^{2}+{\left(Y_{ij}^{gt}-Y_{ij}\right)}^{2}+{\left(Z_{ij}^{gt}-Z_{ij}\right)}^{2}}$$

The average 3D joint error is the overall performance of the hand pose estimate used to evaluate the trained model in the test set.

*The Fraction of Frames:* The fraction of frames is plotted with all joints below a certain threshold, giving the maximum Euclidean distance from the ground truth. If the maximum joint error of the frame is within the distance threshold τ, the frame will be considered good. The fraction of frames over different error thresholds τ is calculated as follows:

(16) $$rate=\frac{1}{N}\sum_{i}1\{\max_{j}\left(\sqrt{{\left(X_{ij}^{gt}-X_{ij}\right)}^{2}+{\left(Y_{ij}^{gt}-Y_{ij}\right)}^{2}+{\left(Z_{ij}^{gt}-Z_{ij}\right)}^{2}}\right)\leq \tau \}$$

where 1 denotes an indicator function, and its value equals to 1 if the inequality is correct, otherwise equal to zero.

To verify the effectiveness of our proposed method, we compare it against several existing methods on 3 publicly available datasets (NYU, MSRA, ICVL), including latent random forest (LRF) [20], DeepPrior with refinements (HandsDeep) [38], feedback loop (Feedback) [44], deep hand model (DeepModel) [11], Lie group-based method (Lie-X) [14], multiview CNN (Multiview) [36], 3D-CNN-based method (3DCNN) [4], CrossingNets [41], and region ensemble network with 9×6×6 region setting (REN-9×6×6) [15].

### 5.1. NYU Hand Pose Dataset

The NYU dataset [4] contains more than 72,000 training images and 8000 test images. The entire dataset is captured using structured light sensors. Thus, deep hand images have missing values and noise, making accurate hand pose estimation more difficult to achieve. For each frame of the depth image, it is shot from three different angles through three Kinect cameras. In the training sample, all depth images contain only one user object (Jonathan Tompson). In the test sample, there are two user objects (Murphy Stein and Jonathan Tompson). To compare with other excellent methods, the depth image acquired by a single Kinect camera and the 14 joints of the human hand are only adopted for comparative analysis, with the same established evaluation metrics [4,14,15,41].

**Figure 2:**
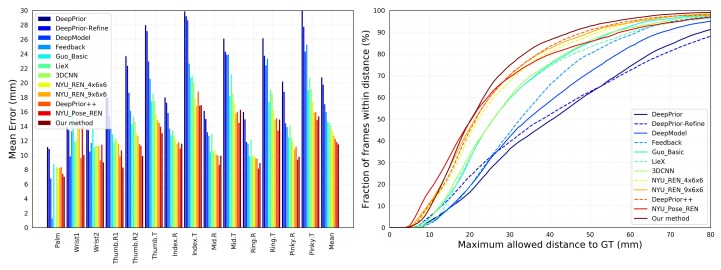
Our approach compares the latest benchmarks on NYU dataset. **Left**: Each joint error. **Right**: The fraction of frames over different maximum Euclidean distance error thresholds. The large area below the curve indicates better results. Our proposed method performs best in the graph of discriminating methods. (Best viewed on screen).

As shown in Table 1, the developed method is compared with [4,10,11,12,13,14,15,16,17,37,38,42,44] by the above two metrics. In Figure 2, the left side shows the average error for each hand joint, and the right graph shows the percentage of frames at different maximum error thresholds. The accuracy of the developed method is higher than the work similar to Oberweger et al. [44]. Meanwhile, the proposed method accomplishes the existing accuracy. When the threshold of the abscissa is less than 20 mm, the Pose-REN [17] curve is always higher than the developed method, which means that there are more frames with an error of less than 20 mm. However, when the abscissa threshold is above 20, the curve is at the top of all other methods. In other words, the larger the area is under the curve, the higher the accuracy at the hand pose estimation.

Our results are shown in Table 1 with a comparison of existing methods. We compare the proposed method with several related methods [38,44], and the results are significantly better than other methods. The most significant advantage of the proposed approach is to increase training samples by generating more depth hand images and reducing over-fitting of the model to improve the accuracy of human pose estimation. We further compare the overall average 3D joint error in Table 1. The developed method obtains 0.4 mm reduction of the average 3D joint error, compared with the current best performance by Pose-REN [17].

### 5.2. MSRA Hand Pose Dataset

The MSRA Gesture Dataset [19] contains approximately 76,500 depth frames captured by Intel Creative Interactive Camera. This dataset includes sequences from 9 different subjects. We perform the leave-one-way cross-validation, which means we train eight different subjects and evaluate the remaining subjects. Meanwhile, we follow the standard established evaluation protocol. The hand annotation consists of 21 joints, each with four joints in the finger and one palm in the palm. This dataset has considerable viewpoint changes. The viewpoint variation makes it a somewhat challenging dataset.

**Figure 3:**
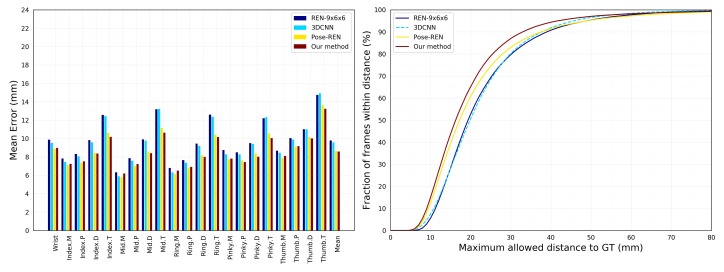
Our approach compares the latest benchmarks on MSRA dataset. **Left**: Each joint error. **Right**: The fraction of frames over different maximum Euclidean distance error thresholds. The large area below the curve indicates better results. Our proposed method performs best in the graph of discriminating methods. (Best viewed on screen).

As shown in Table 2, we compare several state of the art methods [15,16,17,18,19,36,41] on the established protocols including the joint average error and the fraction of frame over different error threshold. Compared with Wang et al. [15] (REN-9×6×6), Chen et al. (Pose-REN) [17], and Wan et al. [41] (CrossingNets), each hand joint average error of the proposed method is lower than that of other methods in the left side of Figure 3. At different threshold errors in the right side of Figure 3, the percentage of frames that satisfy the error threshold is higher than other methods.

**Figure 4:**
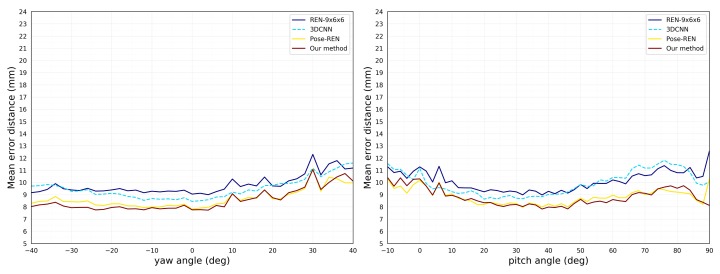
Comparison of mean error distance over different yaw (**left**) and pitch (**right**) viewpoint angles on MSRA dataset. (Best viewed on screen).

Our approach performs best in all assessment methods. Based on the previous evaluation metrics, we also calculated the joint average error distributed over the yaw and pitch angles, as shown in Figure 4. The method we propose has fewer errors at all angles than other methods. It should be noted that when the yaw angle is relatively small ($\left[-40^{\circ },10^{\circ }\right]$), the proposed method will get a relatively small error. As the viewpoint becomes larger ($\left[10^{\circ },40^{\circ }\right]$), the performance of the proposed method is degraded. In the meantime, when the pitch angle is relatively small ($\left[-10^{\circ },40^{\circ }\right]$), the performance of the proposed method is not high. When the viewpoint becomes larger ($\left[40^{\circ },90^{\circ }\right]$), the mean error of the proposed method will decrease slowly.

These results demonstrate that our approach is more robust to viewpoint changes. When the threshold error is greater than 60 mm, the percentage of frames that satisfy the error threshold in our method is slightly lower than that of Pose-REN [17]. This is primarily because the hand pose of the depth image has the inevitable annotated error. However, by comparing the average error of each joint with other methods, our method achieves the best performance in all the comparison methods. Compared to the best performance available with Pose-REN [17], our method achieves a 0.20 mm reduction of the average 3D joint error.

### 5.3. ICVL Hand Posture Dataset

The ICVL hand pose dataset [20] contains over 18k training depth frames, using Intel’s Creative Interactive Gesture Camera, which comprises various hand poses. There are the 1596 test depth frames, containing 702 samples for subject A and 894 samples for subject B. The annotated hand pose has 16 joints, consisting of three joints in each finger, and one joint in the palm. The depth image quality is very high, almost no depth values are lost, the depth image outline is sharp, and there is almost no noise. Though the authors provide different artificial rotation training samples, further data augmentation is needed. Compared to other datasets, the hand pose angle variability is limited, and the annotations are considered inaccurate.

**Figure 5:**
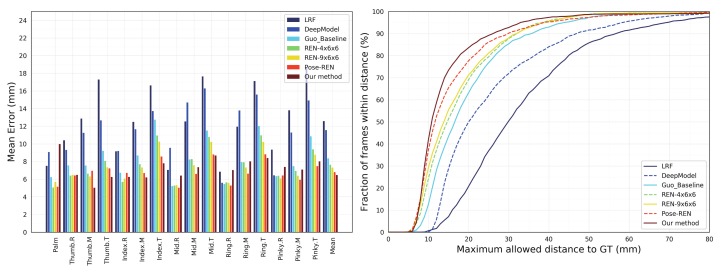
Our approach compares the latest benchmarks on ICVL dataset. **Left**: Each joint error. **Right**: The fraction of frames over different maximum Euclidean distance error thresholds. The large area below the curve indicates better results. Our proposed method performs best in the graph of discriminating methods. (Best viewed on screen).

As shown in Table 3, we compare the proposed method with [10,11,15,16,17,20,37,38,41,47]. Results in Figure 5 demonstrate that the proposed approach greatly outperforms all other methods. Compared with Pose-REN [17], our method decreases the average 3D joint error by 0.34 mm, but the fraction of frames is higher than all other methods between 0 mm and 50 mm.

## 6. Ablation Study

### 6.1. Effects of the Components

In this section, the extensive experiments will be performed to discuss the contribution of different components of our method. The whole model we propose consists of a residual network (ResNet-Hand), a GAN network, and a style-transfer network. To be specific, the function of the residual network is to regress the 3D hand pose estimation. The GAN component is employed to synthesize the smooth depth hand images. The style-transfer network plays a role in transferring the noise of real depth images to smooth depth image.

Table 4 shows that the effects of the different components are numerically calculated on NYU, MSRA, and ICVL, respectively. On NYU dataset, the results are achieved without the use of synthetic images for training. The average 3D joint errors of ResNet-Hand are 13.34 mm, 9.41 mm, and 7.66 mm, respectively. The generator of GAN generates the depth image to decrease the error by 0.45 mm, 0.49 mm, 0.83 mm. Based on the GAN structure, the style transfer achieves 1.49 mm, 0.51 mm, and 0.48 mm reduction of the metric error.

**Figure 6:**
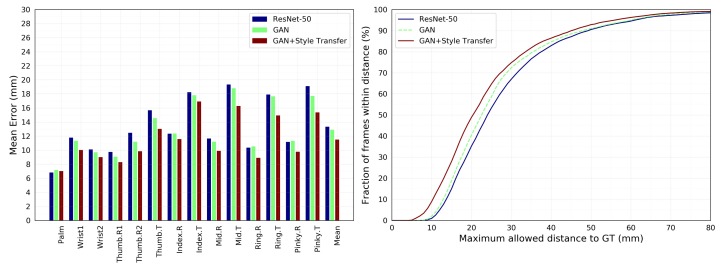
On NYU dataset, the contribution of the different parts to the accuracy are compared. **Left**: Each joint error. **Right**: The fraction of frames over different maximum Euclidean distance error thresholds. The large area below the curve indicates better results. The entire model performs best in the graph. (Best viewed on screen).

In Figure 6, We compare the effect of each part on the entire model, and it is verified that the accuracy of the overall structure is higher than the accuracy of each part.

### 6.2. Visual Results

**Figure 7:**
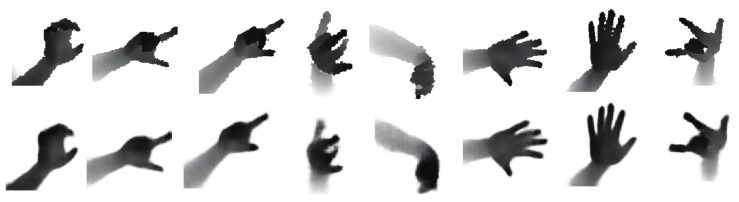
Samples generated by the only GAN for different poses from the test set. **Top:** Ground-truth depth image. **Bottom:** Synthesize depth image using our learned hand model (Best viewed on screen).

**Figure 8:**
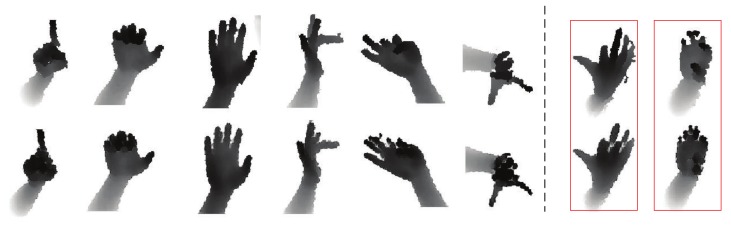
Samples generated by the GAN and style-transfer network for different poses from the test set. **Top:** Ground-truth depth image. **Bottom:** Synthetic depth image using our learned hand model. (Best viewed on screen).

The synthetic images that are largely similar to raw images can be generated, with GAN network and style-transfer structure. We have tried GAN network without style-transfer structure, to gain the synthesized image, which is smoother than the raw image, as show in Figure 7. Due to GAN ignoring the noise of real depth images, style transfer is used to extract the contours of the synthetic image and the textures of style image, and then to mix the content and style features to obtain the phantom in Figure 8. Furthermore, it can be empirically observed that style structure eliminates the shadow of the image background.

A depth hand image generated by using only GAN on the NYU dataset is shown in Figure 7, in which the first row exhibits the ground-truth depth image and the second row displays the synthesized depth hand image. Converting the hand pose information into the depth images only through GAN, the smooth depth hand images can be generated with less hand noise, since the ground-truth depth image generated by the depth camera is noisy, primarily due to dust, diffuse reflection, and illumination changes in the air.

The synthesized depth hand images are shown in Figure 8 using GAN and style-transfer network from the test set in NYU, where the first row exhibits the ground-truth depth images, and the second row displays the synthesized depth hand image. The comparison of the smooth depth hand image with the real one suggests that the style feature of synthesized image is controlled by its specific style image. Furthermore, in Figure 8, the left side of the black dotted line represents the depth of the human hand image with high quality, and the two red rectangles represent the ground-truth depth image and synthesized image of low quality, respectively. In the third column, the real depth of the hand image is compared with the synthetic hand image, the background of real one has shadow as the hand image is extracted by a specific threshold on the original image. However, the background of the synthesized image is clear, so it is proved that our proposed method can eliminate the errors due to the standard method (depth threshold) of extracting the hand image.

As discussed above, the effects of parts in the model are numerically calculated on each dataset. It demonstrates that the synthetic images effectively increase the number of samples and improves the pose estimation accuracy.

**Figure 9:**
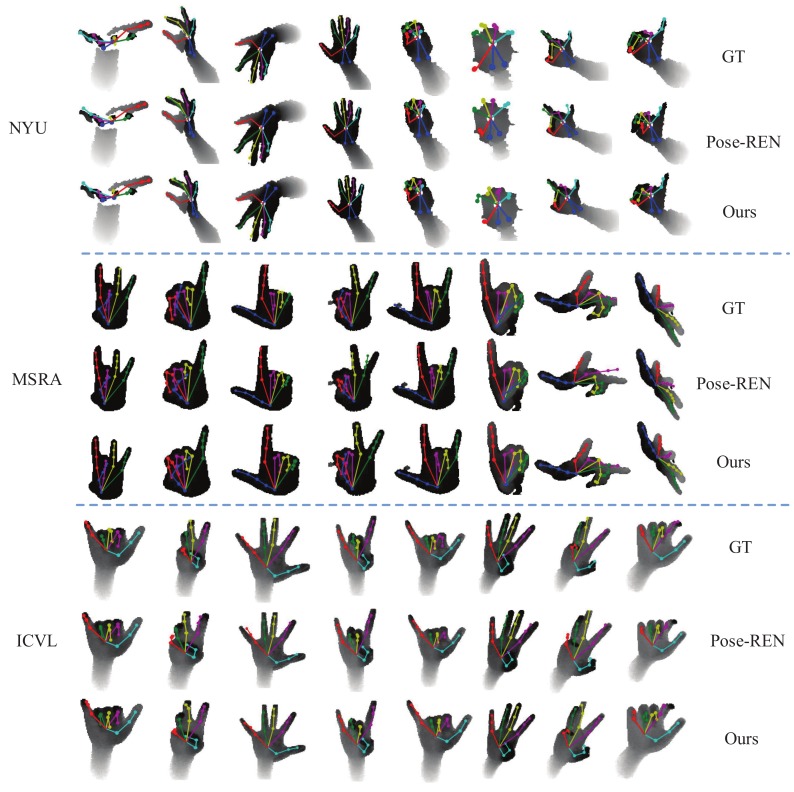
Qualitative results. For each dataset, three rows show the results from pose-guided structured region ensemble network (Pose-REN) [17], our method (Ours) and ground truth (GT), respectively. (Best viewed on screen).

Some qualitative results on three datasets are shown in Figure 9. For each dataset, the first row represents the ground-truth depth hand image, the second row shows the results of Pose-REN [17], the third row is our proposed method. Our proposed method obtains the final estimated results projected into depth hand image on datasets NYU, MSRA, and ICVL. It can be seen that our method performs better than Pose-REN even in some challenging samples.

## 7. Conclusions

In this paper, a novel data-driven approach is developed to generate depth hand image given ground-truth hand poses and to model the statistical relationships of 3D hand poses and corresponding depth images using the generative model. The synthesized images are realistic-looking, having been shown to boost hand pose estimation performance when used as training images. Moreover, the model can enlarge the number of depth hand images to avoid model over-fitting. The proposed approach is evaluated and analyzed on three publicly available datasets, respectively. Then it is demonstrated that the developed method outperforms the existing algorithms. Subsequent work will investigate the human pose datasets and the related tasks.

## Figures and Tables

**Table:** 

**Table 1:** On the NYU dataset [4], we compare the proposed approach to the state of the art. It proves that our method has better results by the evaluation metric of the average 3D joint error in the table below.

Method	Average 3D Error
Bouchacourt et al. [42] (DISCO)	20.7 mm
Oberweger et al. [38] (DeepPrior)	19.8 mm
Deng et al. [10] (Hand3D)	17.6 mm
Zhou et al. [11] (DeepModel)	17.04 mm
Fourure et al. [12] (JTSC)	16.8 mm
Oberweger et al. [44] (Feedback)	16.2 mm
Neverova et al. [13]	14.9 mm
Xu et al. [14] (Lie-X)	14.5 mm
Ge et al. [6] (3DCNN)	14.1 mm
Baek et al. [43]	14.1 mm
Guo et al. [37] (REN-4×6×6)	13.39 mm
Wang et al. [15] (REN-9×6×6)	12.69 mm
Oberweger et al. [16] (DeepPrior++)	12.24 mm
Chen et al. [17] (Pose-REN)	11.81 mm
**This work**	**11.40 mm**

**Table 2:** On the MSRA dataset [19], we compare the proposed approach to the state of the art. It proves that our method has better results by the evaluation metric of the average 3D joint error in the table below.

Method	Average 3D Error
Sun et al. [19] (HPR)	15.2 mm
Yang et al. [18] (Cls-Guided)	13.7 mm
Ge et al. [36] (MultiView)	13.2 mm
Baek et al. [43]	12.5 mm
Wan et al. [41] (CrossingNets)	12.2 mm
Wang et al. [15] (REN-9×6×6)	9.7 mm
Oberweger et al. [16] (DeepPrior++)	9.5 mm
Chen et al.(Pose-REN) [17]	8.65 mm
**This work**	**8.41 mm**

**Table 3:** On the ICVL dataset [20], we compare the proposed approach to the state of the art. It proves that our method has better results by the evaluation metric of the average 3D joint error in the table below.

Method	Average 3D Error
Tang et al. [20] (LRF)	12.6 mm
Zhou et al. [11] (DeepModel)	11.56 mm
Deng et al. [10] (Hand3D)	10.9 mm
Krejov et al. [47] (CDO)	10.5 mm
Oberweger et al. [38] (DeepPrior)	10.4 mm
Wan et al. [41] (CrossingNets)	10.2 mm
Oberweger et al. [16] (DeepPrior++)	8.1 mm
Baek et al. [43]	8.5 mm
Guo et al. [37] (REN-4×6×6)	7.63 mm
Wang et al. [15] (REN-9×6×6)	7.31 mm
Chen et al. [17] (Pose-REN)	6.79 mm
**This work**	**6.45 mm**

**Table 4:** On the NYU, MSRA, and ICVL dataset, the effects of the different parts to accuracy are compared. It proves that the entire model has better results with the average 3D joint error metric in the table below.

Method	ResNet-Hand	GAN	GAN+Style Transfer
NYU [4]	13.34 mm	12.89 mm	11.40 mm
MSRA [19]	9.41 mm	8.92 mm	8.41 mm
ICVL [20]	7.66 mm	6.83 mm	6.45 mm
